# Escitalopram Ameliorates Cognitive Impairment in D-Galactose-Injected Ovariectomized Rats: Modulation of JNK, GSK-3β, and ERK Signalling Pathways

**DOI:** 10.1038/s41598-019-46558-1

**Published:** 2019-07-11

**Authors:** Weam W. Ibrahim, Noha F. Abdelkader, Hesham M. Ismail, Mahmoud M. Khattab

**Affiliations:** 1 Department of Pharmacology & Toxicology, Faculty of Pharmacy, Cairo University, Cairo, Egypt; 2 Department of Pharmaceutical Biochemistry, School of Pharmaceutical Sciences, University of Geneva, Geneva, Switzerland

**Keywords:** Neurodegeneration, Alzheimer's disease

## Abstract

Though selective serotonin reuptake inhibitors (SSRIs) have been found to increase cognitive performance in some studies on patients and animal models of Alzheimer’s disease (AD), other studies have reported contradictory results, and the mechanism of action has not been fully described. This study aimed to examine the effect of escitalopram, an SSRI, in an experimental model of AD and to determine the involved intracellular signalling pathways. Ovariectomized rats were administered D-galactose (150 mg/kg/day, i.p) over ten weeks to induce AD. Treatment with escitalopram (10 mg/kg/day, p.o) for four weeks, starting from the 7^th^ week of D-galactose injection, enhanced memory performance and attenuated associated histopathological changes. Escitalopram reduced hippocampal amyloid β 42, β-secretase, and p-tau, while increasing α-secretase levels. Furthermore, it decreased tumor necrosis factor-α, nuclear factor-kappa B p65, and NADPH oxidase, while enhancing brain-derived neurotrophic factor, phospho-cAMP response element binding protein, and synaptophysin levels. Moreover, escitalopram diminished the protein expression of the phosphorylated forms of c-Jun N-terminal kinase (JNK)/c-Jun, while increasing those of phosphoinositide 3-kinase (PI3K), protein kinase B (Akt), glycogen synthase kinase-3β (GSK-3β), extracellular signal-regulated kinase (ERK) and its upstream kinases MEK and Raf-1. In conclusion, escitalopram ameliorated D-galactose/ovariectomy-induced AD-like features through modulation of PI3K/Akt/GSK-3β, Raf-1/MEK/ERK, and JNK/c-Jun pathways.

## Introduction

Alzheimer’s disease (AD) is the most prevalent neurodegenerative disorder, accounting for up to 80% of dementia cases. Its main clinical manifestations are progressive memory impairment, cognitive deficit, and behavioural disturbances, which severely reduce patients’ quality of life and increase the socio-economic burden^1^. The principal neuropathological characteristics of AD include extracellular senile plaques of amyloid β (Aβ) peptides and intracellular neurofibrillary tangles (NFTs) of hyper-phosphorylated tau (p-tau) proteins. Moreover, AD is accompanied by extensive inflammation and oxidative stress with subsequent synaptic dysfunction as well as profound neurodegeneration in discrete brain regions, including the cortex and hippocampus^1,2^.

Notably, women possess a significantly greater risk of experiencing AD with earlier onset and faster progression than men. The Alzheimer’s Association has estimated that women account for about two-thirds of Americans with AD aged ≥65 years^3^. This divergence is believed to be due to postmenopausal decreases in ovarian estrogen production in women. Indeed, estrogen exerts various neuroprotective effects on the central nervous system, including modulation of the basal forebrain cholinergic system^4^, along with antioxidant, neurotrophic, and anti-inflammatory effects^5^.

Prior studies have indicated that long-term administration of D-galactose (D-gal) in rats or mice results in excessive production of advanced glycation end-products and reactive oxygen species (ROS), and ultimately in cognitive decline^6,7^. Further investigations have revealed that D-gal-injected rodents exhibit several features of brain ageing, including reduced hippocampal neurogenesis, disrupted synaptic plasticity, forebrain cholinergic loss, and Aβ accumulation^7,8^. Likewise, ovariectomy (OVX), a widely used model of menopause, has been reported to impair the learning and memory capacity of female rats, induce neuroinflammation, and reduce neurotrophic factor levels^9^. Previous studies have indicated that estrogen deprivation and D-gal administration act synergistically to accelerate the pathophysiological course of AD^10^. Therefore, OVX along with D-gal injection represents a perfect AD experimental model to mimic the behavioural, neurochemical, and pathological alterations in AD.

Serotonin (5-hydroxytryptamine; 5-HT) is a key neuromodulator implicated in many complex physiological and behavioural functions, such as mood, sleep, appetite, sexual behaviour, thermoregulation, aggression, and stress responses. Increasing evidence has shown that 5-HT also plays a pivotal role in higher cognitive processes, such as learning and memory^11^; however, the mechanism underlying this function remains unclear. Previous autopsy studies have shown pronounced decreases in 5-HT levels and in the numbers of serotonergic neurons in the median and dorsal raphe nuclei of AD patients with copious localization of Aβ deposits in these areas^12,13^. Further imaging experiments have also identified profound alterations in serotonergic receptor density in ageing individuals and AD patients^14^. Moreover, cortical 5-HT levels inversely correlate with NFTs number, proposing that disease development is associated with impairment of the serotonergic system^15^. These previously described studies have indicated that the serotonergic system is markedly affected in AD brain. Taken together, it is hypothesized that selective serotonin reuptake inhibitors (SSRIs), a commonly prescribed class of antidepressants, may provide new selective treatments for cognitive disorders such as AD.

Some clinical and experimental investigations have verified a favourable cognitive enhancing effect of SSRIs^16,17^. In addition, SSRIs have been reported to promote the levels of neurotrophins including brain-derived neurotrophic factor (BDNF), enhance the hippocampal neurogenesis, and decrease Aβ levels^18,19^. Escitalopram (Esc), one of the SSRIs, has also been shown to ameliorate tau hyper-phosphorylation and spatial memory deficits induced by forskolin in rats^17^. However, other studies have documented conflicting results^20,21^. Furthermore, the molecular mechanisms underlying the effects of SSRIs on the neuropathological features of AD, including tau phosphorylation and Aβ deposition, warrant further investigation.

Thus, the present study aimed to elucidate the therapeutic potential of Esc in attenuating learning and memory deficits established by OVX in combination with chronic D-gal injection in female rats. Moreover, the pro-survival and pro-apoptotic signalling cascades involved in Esc-induced ameliorative effects on AD-like pathological alterations were determined.

## Materials and Methods

### Animals

Three to four-month-old female Wistar rats (130–190 g) were obtained from the animal facility of the Faculty of Pharmacy, Cairo University, Cairo, Egypt. They were maintained under controlled temperature and humidity with a 12 h day/night cycle. Access to water and food was permitted ad libitum throughout the study.

### Compliance with ethical standards

This study was conducted in accordance with the Guide for the Care and Use of Laboratory Animals published by the US National Institutes of Health (NIH Publication No. 85–23, revised 2011) and was approved by the Ethical Committee for Animal Experimentation of the Faculty of Pharmacy, Cairo University, with permit number PT 2011.

### Drugs and chemicals

Esc and D-gal were obtained from Multi-Apex Pharmaceutical Company (Cairo, Egypt) and Sigma-Aldrich Chemical Co. (St. Louis, MO, USA), respectively, and doses were prepared daily using physiological saline. All other chemicals were of analytical grade.

### Experimental design

**Figure 1:**
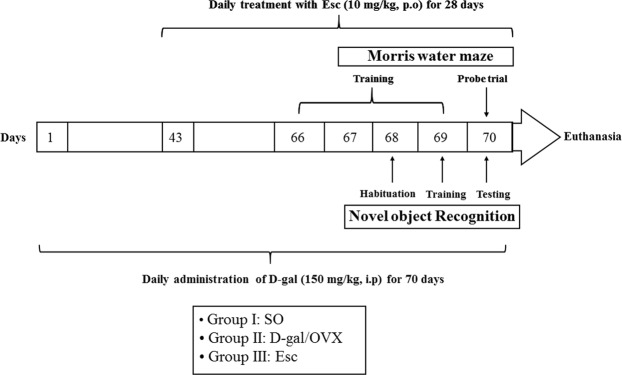
Schematic representation of the experimental design. *SO* sham operation, *D-gal* D-galactose, *OVX* ovariectomy, *Esc* escitalopram.

Sixty female rats were randomly allocated into three groups of 20 rats each. In group I (the sham operation [SO] group), the rats were subjected to SO and given an appropriate volume of saline solution intraperitoneally. In group II (the D-gal/OVX group), the rats were subjected to bilateral OVX and given daily intraperitoneal doses of D-gal (150 mg/kg)^10^ for 10 successive weeks. In group III (the Esc group), D-gal/OVX-subjected rats were treated daily with oral doses of Esc (10 mg/kg)^17^ for 4 weeks starting from the 7^th^ week of D-gal injection. Five days before the end of the experiment, the rats were subjected to Morris water maze (MWM) and novel object recognition (NOR) tests. MWM training was performed on 4 successive days (days 66–69), and on the 5^th^ day (day 70), the probe trial was conducted. NOR was carried out on 3 consecutive days (days 68–70); the habituation phase on day 68, the training phase on day 69, and the testing phase on day 70 (Fig. 1).

### Brain processing

Twenty-four hours following the MWM probe trial and NOR test, the rats were euthanized under anaesthesia by cervical dislocation; afterwards, the brains were quickly excised. The isolated brains from each group were divided into three sets. The brains of the first set (n = 3 per group) were kept in 10% formalin for histological evaluation of the hippocampal area. For the other sets, the hippocampus was dissected from each brain and stored at −80 °C. The hippocampi of the second set (n = 7 per group) were homogenized (10% w/v) in ice-cold saline for determination of Aβ42, β-secretase, BDNF, tumor necrosis factor-α (TNF-α), and nuclear factor-kappa B p65 (NF-κB p65) levels using ELISA kits. In the third set (n = 10 per group), the hippocampi were used for measurement of synaptophysin, nicotinamide adenine dinucleotide phosphate (NADPH) oxidase (NOX) 1, and α-secretase using quantitative RT-PCR as well as for estimation of the protein levels of p-tau, phospho-cAMP response element binding protein (p-CREB), and the phosphorylated (p-) forms of phosphoinositide 3-kinase (PI3K), protein kinase B (Akt), glycogen synthase kinase-3β (GSK-3β), extracellular signal-regulated kinase (ERK) 1/2, ERK kinase (MEK) 1/2, ERK kinase kinase (Raf-1), c-Jun N-terminal kinase (JNK), and c-Jun using Western blot analysis.

### Ovariectomy

Rats were anaesthetized using intraperitoneal injections of ketamine (50 mg/kg) and xylazine (10 mg/kg). Using sterile surgical techniques, bilateral OVX was conducted by making a small incision between the hip and the last rib on each lateral side of the abdomen. The ovaries and fallopian tube were clamped, ligated, and then excised. The muscle and skin layers were sutured. Sham surgery was carried out in the same way but without resection of the ovaries. After surgery, all rats were injected subcutaneously with 0.1 ml of each of cefotaxime (100 mg/ml) and diclofenac sodium to enhance the healing process, and they were allowed access to soy-free chow to exclude any effects of phytosteroids in the diet.

### Behavioural assessment

#### Morris water maze

In rodents, spatial learning and memory are commonly investigated using the MWM test. The maze consisted of a large circular pool (60 cm high and 150 cm in diameter) that was filled to a depth of 40 cm with water maintained at room temperature. The pool was divided arbitrarily into four equal quadrants using two threads perpendicular to each other that were fixed to the edge of the pool. A platform (8 cm in diameter) was placed in the centre of a specific quadrant just below the water surface. In the acquisition phase, rats were subjected daily to 2 training sessions of 120 s each for 4 consecutive days. During each training session, the animals were left free to find the hidden platform in the target quadrant. If the rat succeeded in finding the hidden platform within the designated 120 s, it was permitted to remain on the platform for 10 s. However, if the animal failed to locate the platform during the allocated time, it was gently guided to reach the platform and was placed on it for 30 s. The escape latency was calculated as the average of the total time taken to find the platform in the two training sessions on each day of the acquisition phase and was used as a measure of spatial learning progress. On the 5^th^ day, the rats were subjected to a probe test (retrieval trial) in which the platform was removed and each rat was placed for 120 s in the pool starting from the quadrant opposite to the platform quadrant. The time spent by each animal in the target quadrant searching for the removed platform was considered an index of retrieval^9,10^.

#### Novel object recognition

The NOR test is based on the native propensity of rodents to show preference for exploring novel objects over familiar ones. The test apparatus was an open box (50 cm wide x 60 cm long x 80 cm high) made of wood with a floor divided by equidistant lines into 12 equal rectangles. The test consisted of three phases performed on three successive days. On the first day, nominated for the habituation phase, the rats were allowed to freely explore the open field in the absence of objects for 5 min. Instances of line crossing and rearing performed in this phase were counted and used as measures of locomotor and exploratory behaviours, respectively. Twenty-four hours later, the familiarization or training phase was conducted by placing each rat into the field, which contained two similar objects in opposite corners 6 cm from the walls. Each rat was allowed to explore the objects for 3 min. On the third day, the test phase was performed; in which one of the two identical objects was removed and substituted with a new one that was different from the objects with which the rats were familiar. Each animal was permitted to explore both objects for 3 min. During the training and testing phases, the floor was covered with sawdust and the objects were carefully cleaned after each tested animal. The exploration time for the familiar or novel object during the test phase was recorded. The discrimination index, which is the difference in the time spent exploring the novel and familiar objects divided by the total exploration time for both objects, was calculated. Moreover, the recognition index, which is the time spent exploring the novel object as a percentage of the total time spent exploring both objects, was estimated^10,22^.

#### Histopathological examination

Brain samples fixed in 10% formalin were embedded in paraffin after gradient dehydration. The obtained paraffin blocks were then sectioned at 3 μm thickness. The brain sections were de-paraffinized, hydrated, and then stained with haematoxylin and eosin (H&E) for histopathological examination under a light microscope. The degree of severity of the hippocampal degenerative changes was scored using a subjective scoring system ranging from 0 to 3, where 0 = absent, 1 = mild, 2 = moderate, and 3 = severe^10^.

#### Biochemical measurements

Determination of hippocampal Aβ42, β-secretase, BDNF, TNF-α, and NF-κB p65 levels using ELISA kits. Using rat-specific ELISA kits, the hippocampal levels of Aβ42 and NF-κB p65 (Elabscience Biotechnology Co., Ltd., Wuhan, Hubei, China, cat. nos. E-EL-R1402 and E-EL-R0674, respectively), as well as those of β-secretase (β-site amyloid cleaving enzyme-1), BDNF, and TNF-α (MyBioSource, San Diego, CA, USA, cat. nos. MBS2512086, MBS355345, and MBS355371, respectively) were assessed according to the manufacturers’ guidelines. The results were expressed as pg/mg protein for Aβ42, BDNF, TNF-α, and NF-κB p65, and as ng/mg protein for β-secretase. The protein content was determined according to the method previously described^23^.

Determination of hippocampal p-tau, p-CREB, p-PI3K, p-Akt, p-GSK-3β, p-JNK, p-c-Jun, p-Raf-1, p-MEK1/2, and p-ERK1/2 protein expression by Western blot analysis. The isolated hippocampi were homogenized in lysis buffer containing 50 mM Tris-HCl (pH 7.4), 10 mM NaF, 2 mM EDTA, 1 mM PMSF, 10 mM β-glycerol phosphate, and protease inhibitor cocktail. The obtained homogenates were centrifuged at 12000 rpm and 4 °C for 20 min and then the protein concentration was determined in lysate aliquots using a Bio-Rad protein assay kit (CA, USA). The protein samples were separated by SDS polyacrylamide gel electrophoresis and transferred onto nitrocellulose membranes using a semi-dry transfer apparatus (Bio-Rad, CA, USA). The membranes were blocked with 5% blocking solution containing non-fat dry milk in Tris-buffered saline with Tween (TBST) for 1 h at 37 °C to prevent non-specific interactions between the antibodies and the nitrocellulose membranes. The membranes were then incubated overnight on a roller shaker at 4 °C with 1:1000 dilutions of the following primary antibodies: anti-β-actin (4967, Cell Signaling Technology, USA), anti-p-PI3K p85 (Tyr458)/p55 (Tyr199) (4228, Cell Signaling Technology, USA), anti-p-Akt (Ser473) (4060, Cell Signaling Technology, USA), anti-p-GSK-3β (Ser9) (5558, Cell Signaling Technology, USA), anti-p-JNK (Thr183/Tyr185) (44–682 G, Thermo Fisher Scientific, USA), anti-p-c-Jun (Ser73) (PA5-17879, Thermo Fisher Scientific, USA), anti-p-c-Raf (Ser338) (MA5-15176, Thermo Fisher Scientific, USA), anti-p-MEK1/2 (Ser217/221) (MA5-15016, Thermo Fisher Scientific, USA), anti-p-ERK1/2 (Thr202/Tyr204) (36–8800, Thermo Fisher Scientific, USA), anti-p-tau (Ser404) (44–758 G, Thermo Fisher Scientific, USA), and anti-p-CREB (Ser133) (PA1-4619, Thermo Fisher Scientific, USA). After washing in TBST, the membranes were probed with the secondary antibody, HRP-conjugated goat anti-mouse immunoglobulin (Dianova, Hamburg, Germany). Finally, the protein bands were developed using an enhanced chemiluminescence substrate reaction (Amersham Biosciences, Arlington Heights, IL, USA). The protein band intensities were quantified by densitometric analysis using a scanning laser densitometer (Biomed Instrument, Inc., USA). The results were presented as arbitrary units relative to the intensity of the corresponding β-actin bands.

Determination of hippocampal synaptophysin, NOX1, and α-secretase gene expression by quantitative RT-PCR. Total RNA was extracted from hippocampal samples using an RNeasy Mini Kit (Qiagen, Venlo, Netherlands) in compliance with the manufacturer’s instructions. Any residual DNA was removed by DNase supplied with the kit. The quantity of the isolated RNA in each sample was determined at 260 nm using a NanoDrop ND-1000 spectrophotometer (Thermo Fisher Scientific, Waltham, USA), and RNA purity was assessed based on the 260/280 nm absorption ratio. For cDNA synthesis, a reverse transcription system (Promega, Leiden, Netherlands) was used according to the manufacturer’s protocol. The RNA was incubated with MgCl_2_ (25 mM), RTase buffer (10×), a dNTP mixture (10 mM), oligo d (t) primers, RNase inhibitor (20 U), and AMV reverse transcriptase (20 U/μl) at 42 °C for 1 h.

The expression levels of the synaptophysin, NOX1, and α-secretase (a disintegrin and metallopeptidase domain 10) genes were analysed by quantitative RT-PCR using SYBR Green PCR Master Mix (Applied Biosystems, CA, USA) with an ABI PRISM 7500 Fast Sequence Detection System (Applied Biosystems, CA, USA) and the associated quantification software (Applied Biosystems, CA, USA). The sequences of the primers used are listed in Table 1. β-Actin was used as the housekeeping gene. The relative expression of the target genes was obtained using the comparative C_T_ (ΔΔC_T_) method. ΔC_T_ was calculated by subtracting the C_T_ value of reference gene (β-actin) from that of each target gene, whereas ΔΔC_T_ was obtained by subtracting the ΔC_T_ of the calibrator sample (SO group) from that of the test sample (D-gal/OVX or Esc group). The relative expression was calculated from the 2^−ΔΔCT^ formula^24^.

**Table 1:** The primers sequences.

mRNA species	Accession number	Primer sequence (5′‒3′)
Synaptophysin	NM_012664	F: CTTTCTGGCTACAGCCGTGTTCG R: GTTCCCTGTCTGGCGGCACATG
NOX1	NM_053683.1	F: TCTTGCTGGTTGACACTTGC R: TATGGGAGTGGGAATCTTGG
α-secretase	NM_007399.4	F: AGTAGTAATCCAAAGTTGCCG R: GTGTCCCATTTGATAACTCTCTC
β-actin	NM_031144.3	F: TATCCTGGCCTCACTGTCCA R: AACGCAGCTCAGTAACAGTC

*NOX* nicotinamide adenine dinucleotide phosphate oxidase.

### Statistical analysis

Most results were analysed using one-way ANOVA followed by Tukey’s multiple comparisons test and were expressed as the mean ± SD. However, escape latency during the MWM acquisition phase was analysed by two-way ANOVA followed by Tukey’s multiple comparisons test. Furthermore, line crossings, rearing, and histopathological scores were analysed using Kruskal-Wallis test followed by Dunn’s multiple comparisons test and were expressed as the median and range. For all statistical tests, the level of significance was set at p < 0.05. GraphPad Prism software (version 6) was used to carry out the statistical analysis.

## Results

### Escitalopram ameliorated cognitive dysfunction induced in D-gal/OVX rats

**Figure 2:**
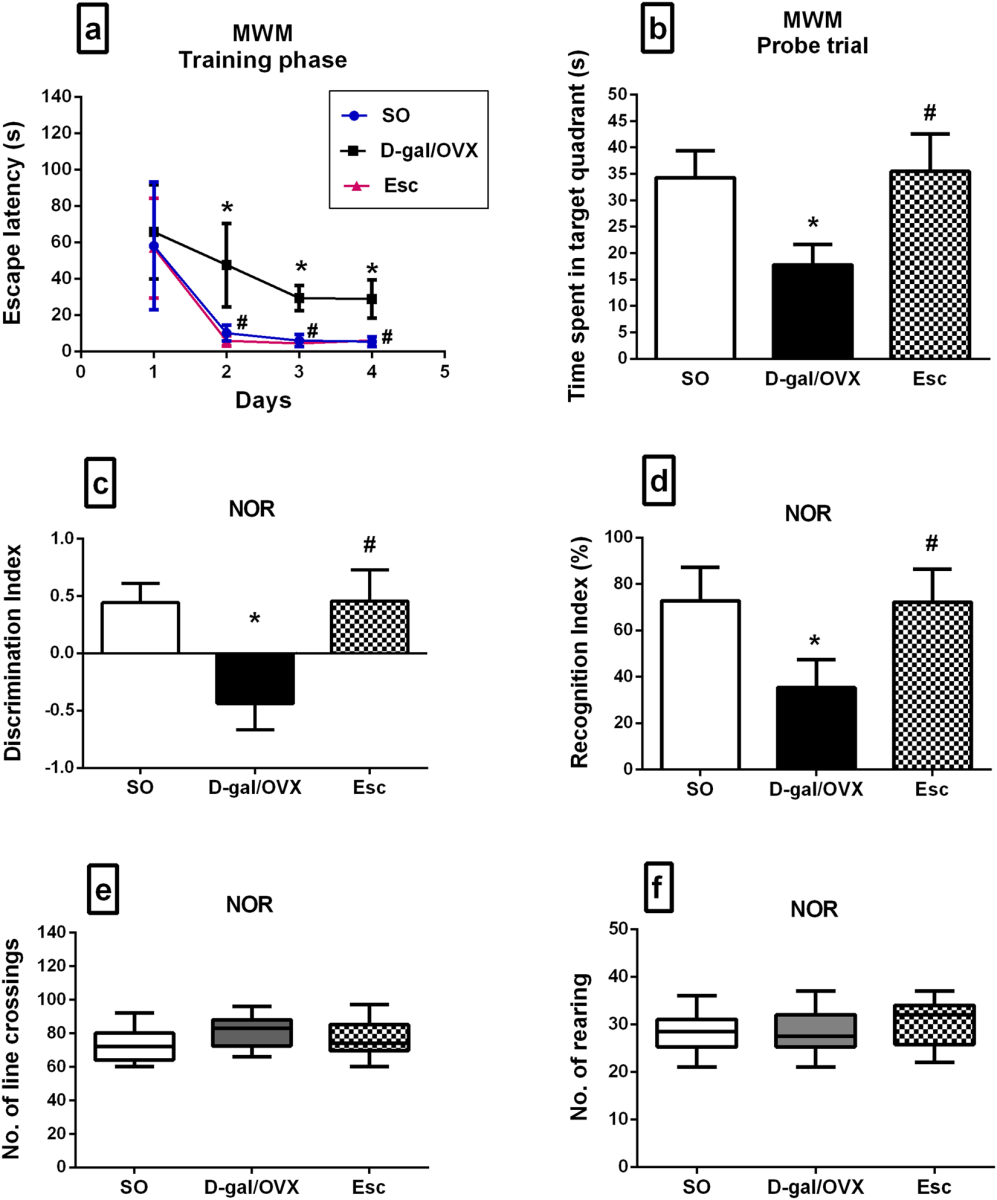
Escitalopram ameliorated cognitive dysfunction induced in D-galactose/ovariectomized rats in the MWM (**a**,**b**) and NOR tests (**c**–**f**). Data were expressed as mean ± SD for a, b, c, and d, and as median and range for e and f (n = 20). * vs SO group, ^**#**^ vs D-gal/OVX group, using one-way ANOVA followed by Tukey’s multiple comparisons test; except for escape latency that was analysed by two-way ANOVA followed by Tukey’s multiple comparisons test, and for line crossings and rearing that were compared using Kruskal-Wallis test followed by Dunn’s multiple comparisons test; p < 0.05. *SO* sham operation, *D-gal* D-galactose, *OVX* ovariectomy, *Esc* escitalopram, *MWM* Morris water maze, *NOR* novel object recognition.

In the MWM test, the D-gal/OVX group displayed markedly greater escape latencies during the second, third, and fourth days of the acquisition phase by approximately 4.7-, 4.9-, and 5.4-fold, respectively, as compared with the SO group (Fig. 2a). On the other hand, Esc treatment enhanced the spatial learning capabilities of D-gal/OVX-subjected rats, as demonstrated by reductions in the escape latency during the last three training days to values that were not significantly different from the normal values (Fig. 2a). In the probe trial, D-gal/OVX rats exhibited a lower index of retrieval, which manifested as less time spent in the target quadrant searching for the removed platform (48%) as compared with SO group, while treatment with Esc returned the index to a normal level (Fig. 2b). In the NOR test, the discrimination index was decreased in D-gal/OVX group by approximately 200% as compared to SO group, whereas Esc-treated rats were able to differentiate between familiar and novel objects and thus exhibited normal discrimination index values (Fig. 2c). Additionally, D-gal/OVX rats spent less time exploring the novel object, as verified by their lower recognition index (51%) as compared with SO rats; however, Esc administration increased the recognition index to values that were not significantly different from the SO group values (Fig. 2d). Furthermore, the numbers of line crossings and rearing, indicative of the rats’ locomotor and exploratory activity, respectively, were not significantly different among the experimental groups; this finding excludes the involvement of motor functions in the rats’ cognitive performance (Fig. 2e,f).

### Escitalopram attenuated histopathological alterations induced in the hippocampi of D-gal/OVX rats

**Figure 3:**
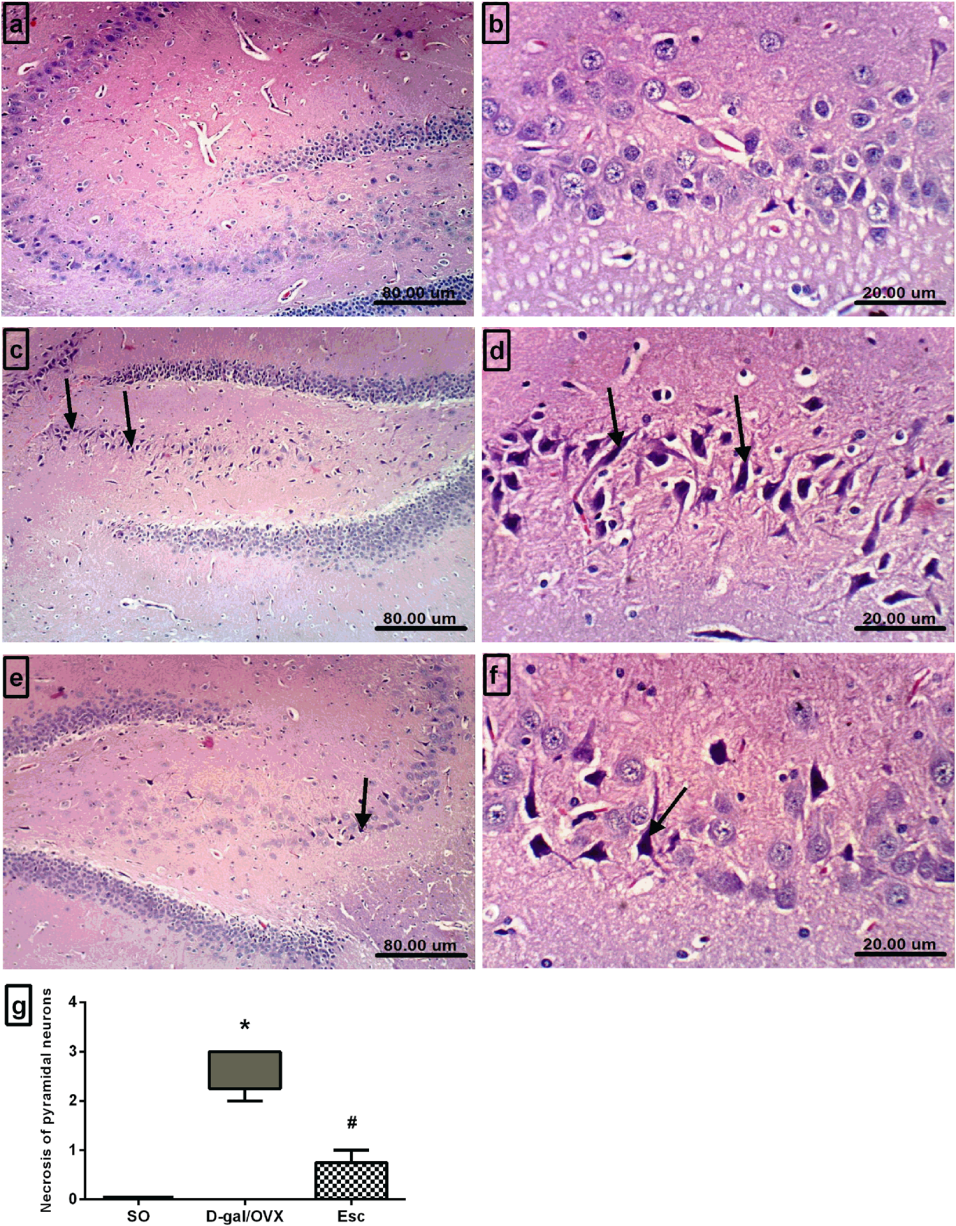
Escitalopram attenuated histopathological alterations induced in the hippocampi of D-galactose/ovariectomized rats. Representative photomicrographs of the hippocampal sections of the SO group **(a**,**b)** demonstrated normal histological appearance, those of the D-gal/OVX group **(c**,**d)** showed degeneration of the pyramidal neurons *(arrows)*, and those of the Esc group **(e**,**f)** revealed marked reductions in the numbers of necrotic neurons *(arrow)* (H&E, 100 × and 400×). **(g)** Histopathological scoring of pyramidal neuron necrosis. Data were expressed as median and range (n = 3). * vs SO group, ^**#**^ vs D-gal/OVX group using Kurskal-Wallis test followed by Dunn’s multiple comparisons test; p < 0.05. *SO* sham operation, *D-gal* D-galactose, *OVX* ovariectomy, *Esc* escitalopram.

The microscopic investigation of the brain sections harvested from the SO animals demonstrated an intact architecture of the hippocampus with normal-appearing neurons (Fig. 3a,b). In contrast, the hippocampi of the D-gal/OVX group revealed severe degenerative alterations and necrosis of pyramidal neurons (Fig. 3c,d), as indicated by the higher histopathological scores in the D-gal/OVX group than in the SO group (Fig. 3g). Treatment of D-gal/OVX-subjected rats with Esc attenuated the pathological degenerative changes, as demonstrated by a pronounced decrease in the number of necrotic neurons (Fig. 3e–g).

### Escitalopram mitigated D-gal/OVX-induced alterations in the PI3K/Akt/GSK-3β signalling pathway in rats

**Figure 4:**
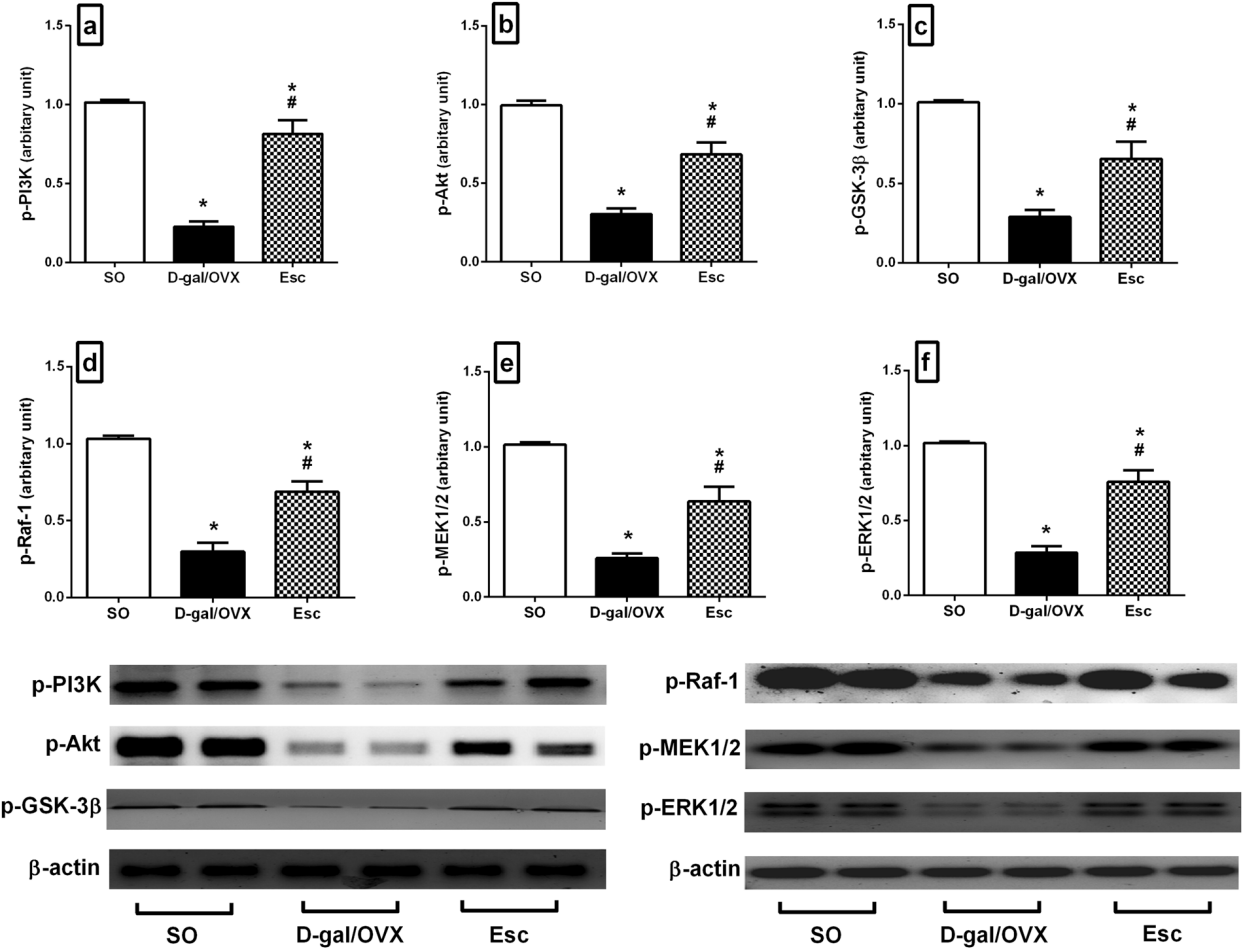
Escitalopram mitigated D-galactose/ovariectomy-induced alterations in the hippocampal protein expression of **(a)** p-PI3K, **(b)** p-Akt, **(c)** p-GSK-3β, **(d)** p-Raf-1, **(e)** p-MEK1/2, and **(f)** p-ERK1/2 in rats. The cropped blots for these proteins were presented relative to that of β-actin, and the uncropped images are available in the Supplementary File. Each bar with a vertical line represents mean ± SD (n= 5). * vs SO group, ^**#**^ vs D-gal/OVX group, using one-way ANOVA followed by Tukey’s multiple comparisons test; p < 0.05. *SO* sham operation, *D-gal* D-galactose, *OVX* ovariectomy, *Esc* escitalopram, *PI3K* phosphoinositide 3-kinase, *Akt* protein kinase B, *GSK-3β* glycogen synthase kinase-3β, *ERK* extracellular signal-regulated kinase.

D-gal/OVX group showed marked declines in the hippocampal protein expression of p-PI3K (78%), p-Akt (69%), and p-GSK-3β (71%) compared to their SO group counterparts (Fig. 4a–c). On the contrary, Esc-treated animals exhibited spike increase in the levels of these proteins by 3.6-fold (p-PI3K), 2.3-fold (p-Akt), and 2.3-fold (p-GSK-3β) compared with D-gal/OVX rats (Fig. 4a–c).

### Escitalopram diminished D-gal/OVX-induced alterations in the Raf-1/MEK/ERK signalling pathway in rats

The hippocampal protein expression levels of p-Raf-1, p-MEK1/2, and p-ERK1/2 were markedly reduced by 71, 75, and 72%, respectively, in D-gal/OVX group compared to SO group (Fig. 4d–f). However, Esc treatment attenuated these reductions by 2.3-fold (p-Raf-1), 2.5-fold (p-MEK1/2), and 2.7-fold (p-ERK1/2) compared to D-gal/OVX group (Fig. 4d–f).

### Escitalopram curbed D-gal/OVX-induced alterations in the JNK/c-Jun signalling pathway in rats

**Figure 5:**
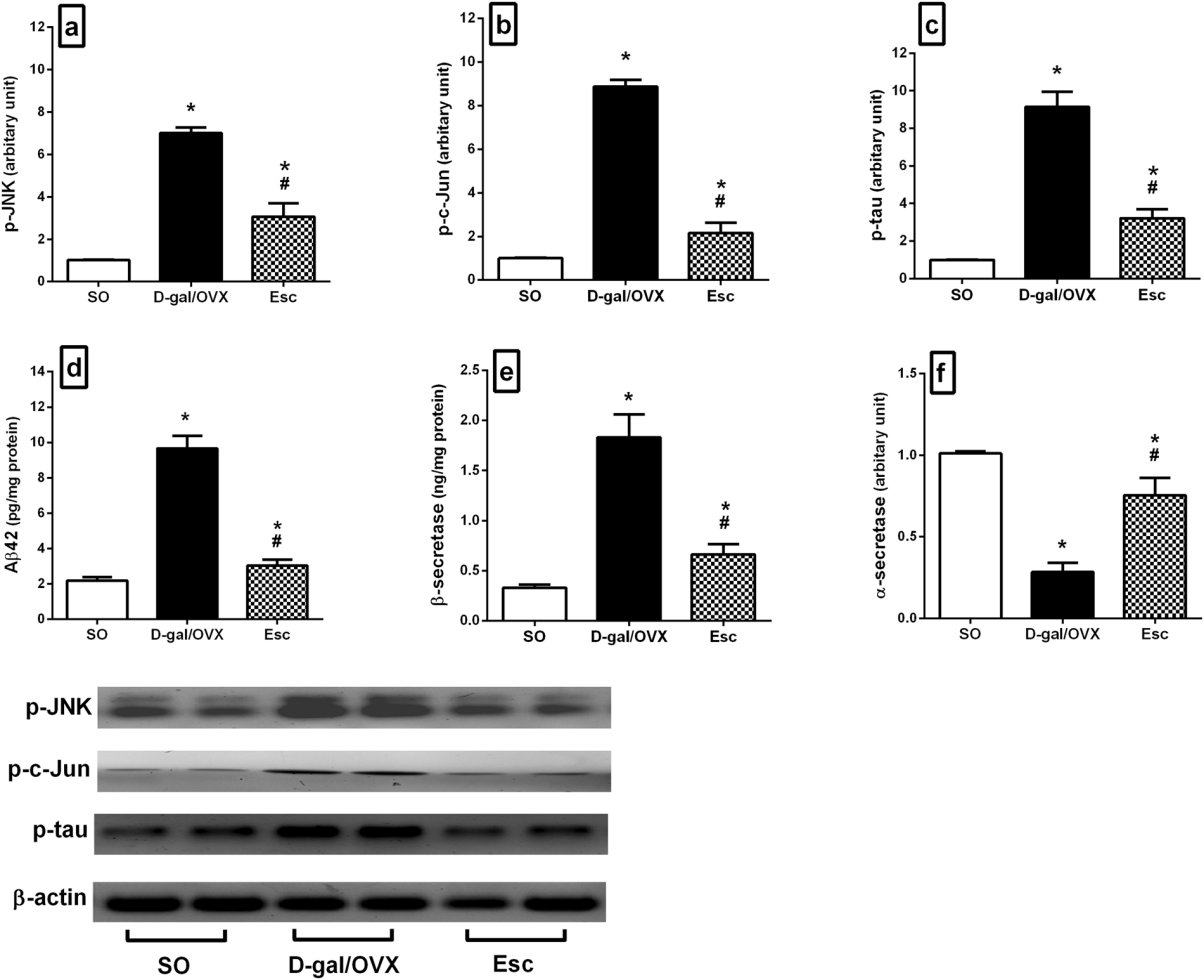
Escitalopram amended D-galactose/ovariectomy-induced alterations in the hippocampal levels of **(a)** p-JNK, **(b)** p-c-Jun, **(c)** p-tau, **(d)** Aβ42, **(e)** β-secretase, and **(f)** α-secretase in rats. The cropped blots of p-JNK, p-c-Jun, and p-tau were presented relative to that of β-actin, and the uncropped images are available in the Supplementary File. Each bar with a vertical line represents mean ± SD (n= 5). * vs SO group, ^**#**^ vs D-gal/OVX group, using one-way ANOVA followed by Tukey’s multiple comparisons test; p < 0.05. *SO* sham operation, *D-gal* D-galactose, *OVX* ovariectomy, *Esc* escitalopram, *JNK* c-Jun N-terminal kinase, *p-tau* phosphorylated tau, *Aβ42* amyloid β 42.

OVX combined with D-gal injection significantly elevated the hippocampal protein expression of p-JNK (6.9-fold) and p-c-Jun (8.9-fold) relative to SO (Fig. 5a,b). These effects were mitigated by Esc, which reduced the levels of p-JNK and p-c-Jun by 56 and 76%, respectively, relative to D-gal/OVX group (Fig. 5a,b).

### Escitalopram amended D-gal/OVX-induced alterations in the hippocampal levels of p-tau, Aβ42, β-secretase, and α-secretase in rats

D-gal administration and OVX induced massive increment in hippocampal Aβ42 (4.4-fold) and β-secretase (5.6-fold) levels, along with an increase in p-tau protein levels (9.1-fold) and a 72% reduction in α-secretase gene expression compared to SO (Fig. 5c–f). Conversely, Esc treatment decreased the levels of p-tau (65%), Aβ42 (69%), and β-secretase (64%) and enhanced the levels of α-secretase (2.7-fold) in D-gal/OVX-subjected rats (Fig. 5c–f).

### Escitalopram curtailed D-gal/OVX-induced alterations in the hippocampal levels of BDNF, p-CREB, and synaptophysin in rats

**Figure 6:**
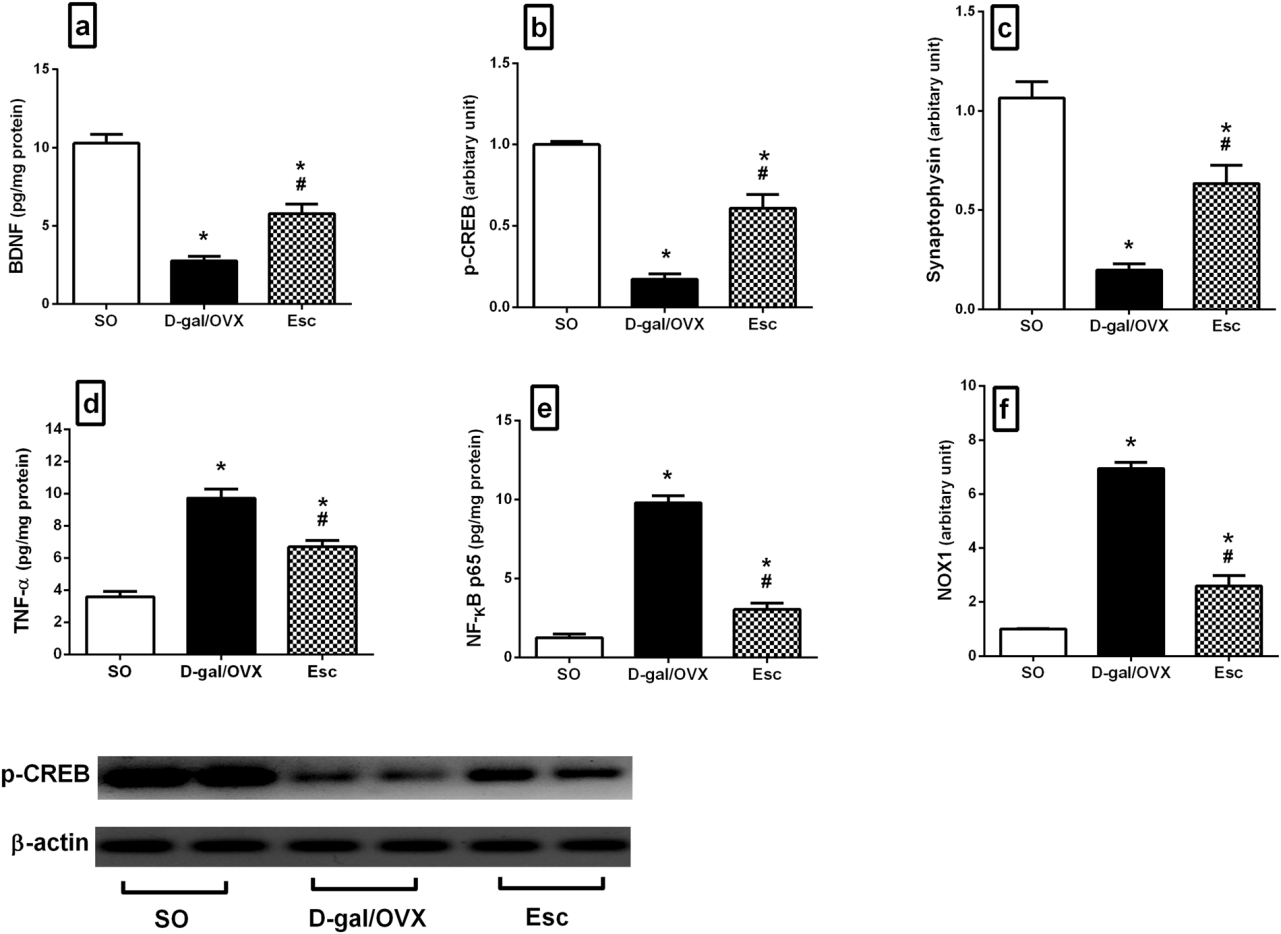
Escitalopram hampered D-galactose/ovariectomy-induced alterations in the hippocampal levels of **(a)** BDNF, **(b)** p-CREB, **(c)** synaptophysin, **(d)** TNF-α, **(e)** NF-κB p65, and **(f)** NOX1 in rats. The cropped blot of p-CREB was presented relative to that of β-actin, and the uncropped images are available in the Supplementary File. Each bar with a vertical line represents mean ± SD (n= 5). * vs SO group, ^**#**^ vs D-gal/OVX group, using one-way ANOVA followed by Tukey’s multiple comparisons test; p < 0.05. *SO* sham operation, *D-gal* D-galactose, *OVX* ovariectomy, *Esc* escitalopram, *BDNF* brain-derived neurotrophic factor, *p-CREB* phospho-cAMP response element binding protein, *TNF-α* tumor necrosis factor-α, *NF-κB p65* nuclear factor-kappa B p65, *NOX* nicotinamide adenine dinucleotide phosphate oxidase.

D-gal/OVX rats exhibited prominent reductions in hippocampal BDNF content and p-CREB protein expression by 73 and 83%, respectively, along with 81% declines in synaptophysin gene expression relative to SO rats (Fig. 6a–c). Esc treatment reversed these diminutions, producing 2.1-fold (BDNF), 3.5-fold (p-CREB), and 3.2-fold (synaptophysin) higher levels than those in the D-gal/OVX group (Fig. 6a–c).

### Escitalopram hampered D-gal/OVX-induced alterations in the hippocampal levels of TNF-α, NF-κB p65, and NOX1 in rats

OVX combined with D-gal administration aggravated inflammatory states and oxidative stress, as reflected by prominent escalation in the hippocampal contents of TNF-α (2.7-fold) and NF-κB p65 (7.9-fold) and an increase in the gene expression of NOX1 (7-fold) in the D-gal/OVX group compared to the SO group (Fig. 6d–f). In contrast, administration of Esc attenuated these elevations, resulting in 31% (TNF-α), 69% (NF-κB p65), and 63% (NOX1) lower levels in the Esc group than in the D-gal/OVX group (Fig. 6d–f).

## Discussion

The present investigation substantiated that Esc could alleviate cognitive dysfunction and slow associated AD-like pathological changes in D-gal/OVX-subjected rats. Esc reduced the gene expression of the ROS-generating enzyme NOX1 in addition to exerting anti-neuroinflammatory and neurotrophic effects. These effects were related, in part, to activation of the pro-survival PI3K/Akt/GSK-3β and Raf-1/MEK/ERK cascades and inhibition of the pro-apoptotic JNK/c-Jun pathway.

In the current investigation, treatment with Esc improved learning and memory processes in the MWM and NOR tests; these effects were not due to altered sensorimotor functions^25^. Previous literatures evaluating the influence of SSRIs on cognitive functions have reported contradictory results. While some reports have shown memory enhancing effects of SSRIs^17^, other studies have reported either null^21^ or even detrimental effect^20^. Such irreconcilable findings may be partly attributable to differences in the maintenance doses of SSRIs or the durations of drug administration.

In the present work, the elevated hippocampal content of Aβ42 in D-gal/OVX rats was associated with reduced α-secretase and enhanced β-secretase levels. Aβ42 is the most aggregative and neurotoxic form of Aβ, which is produced by proteolytic cleavage of amyloid precursor protein (APP) by β- and γ-secretase^26^. β-Secretase cleavage is a vital step for Aβ42 generation, and it has been reported that the activity of β-secretase is increased in AD patient brains and experimental models of AD^27,28^. Alternatively, APP may undergo cleavage via α-secretase within the Aβ sequence, precluding Aβ generation^29^.

In this study, Esc administration repressed D-gal/OVX-induced alterations in α- and β-secretase levels and subsequently decreased Aβ42 levels. It has been postulated that this effect is partly attributed to 5-HT-mediated activation of the ERK signalling pathway^19^. The present study revealed prominent increases in the protein expression of the active phosphorylated forms of ERK1/2 and its upstream kinases Raf-1 and MEK1/2 in response to Esc treatment. In fact, stimulation of 5-HT G-protein-coupled receptors has been found to cause chronological activation of Raf-1, MEK, and ERK^19^. Activated ERK can remain in the cytosol to phosphorylate proteins, altering their function, or translocate into the nucleus to regulate the transcription of various genes. Activated ERK is expected to play a role in reducing Aβ generation; as it negatively regulates the expression and activity of β- and γ-secretase enzymes^30,31^, while enhancing α-secretase activity^32^. Furthermore, ERK has emerged as a focal mediator of neuronal survival and differentiation^33^. Moreover, inhibition of ERK has been reported to impair memory formation and synaptic plasticity^34^. Hence, activation of Raf-1/MEK/ERK by Esc is regarded as a probable mechanism supporting the potential efficacy of Esc against AD.

Downstream mechanisms of Aβ neurotoxicity include tau hyper-phosphorylation^35^. Abnormal hyper-phosphorylation of tau protein increases the resistance of tau to proteolysis and the formation of insoluble paired helical filaments that are deposited as NFTs. Furthermore, hyper-phosphorylation negatively regulates the binding of tau protein to microtubules, leading to microtubule destabilization, axonal transport impairment, and eventually neuronal death^36^. In the current study, augmented phosphorylation of tau protein was detected in the D-gal/OVX group. Aβ provokes tau phosphorylation, most likely via stimulation of several tau-targeting kinases, including GSK-3β and JNK^37,38^. These kinases also exert positive feedback regulation on Aβ production by modulating the expression of β-secretase or phosphorylating APP at Thr668, inducing its proteolysis by β-secretase to generate Aβ^31,39,40^. In the present work, Esc reduced p-tau protein expression in D-gal/OVX-subjected rats; this effect is probably attributed to Esc-induced Aβ suppression^41^ in addition to the inhibitory effects on GSK-3β and JNK reported herein^42,43^.

The current results revealed that Esc administration mitigated D-gal/OVX-induced elevations in p-JNK and p-c-Jun protein levels. Analyses of post-mortem brain samples from AD patients have demonstrated increased p-JNK expression and positive co-localization with Aβ^44,45^. The induced JNK levels could be partly related to the increased levels of Aβ and NOX1 detected herein^46,47^. JNK plays key roles in tau hyper-phosphorylation, Aβ overproduction, and neuronal apoptosis. After its activation by phosphorylation at Thr183 and Tyr185^48^, p-JNK phosphorylates and activates its downstream transcription factor c-Jun at Ser63 and Ser73^49^. p-JNK also triggers the phosphorylation of tau^37^ and APP at Thr668, enhancing the β-secretase-induced cleavage of APP to Aβ^40^. Furthermore, p-JNK phosphorylates and activates pro-apoptotic proteins, causing the activation of caspases, while phosphorylating and inhibiting anti-apoptotic proteins^50^. Likewise, c-Jun participates in AD pathogenesis. It stimulates β-secretase expression, increasing Aβ generation^31^. Additionally, it accumulates within the structures of NFTs, participating partly in tangle maturation in AD^51^. It has also been proposed that p-c-Jun promotes the transcription of NOX to participate in its overexpression^52^. Thus, inhibition of the JNK/c-Jun cascade by Esc is a putative novel mechanism implicated in the potential ability of Esc to hinder AD progression.

Herein, Esc treatment counteracted D-gal/OVX-induced depletions of the hippocampal protein expression of p-PI3K, p-Akt, and p-GSK-3β, causing prominent increases in the levels of these proteins; these findings are consistent with those of a prior report^17^. Activation of PI3K induces the phosphorylation and activation of Akt. The latter is the upstream negative regulator of GSK-3β, as p-Akt phosphorylates and inhibits GSK-3β. GSK-3β activity is abnormally upregulated in AD patients as a result of inactivation of the upstream PI3K/Akt pathway^53^. GSK-3β plays an integral role in AD pathogenesis. It is intimately involved in tau hyper-phosphorylation^38^ and Aβ overproduction through APP phosphorylation at Thr668, which triggers its β-secretase-mediated proteolysis to form Aβ^39^. GSK-3 also promotes the differentiation and migration of inflammatory cells as well as the release of pro-inflammatory cytokines^54^. In addition, it prevents the induction of long-term potentiation, producing learning and memory impairments^55^. Furthermore, GSK-3 reduces the synthesis of acetylcholine^56^ and modulates key steps of apoptotic signalling pathways^57^. Hence, GSK-3β deactivation by Esc serves as another plausible mechanism involved in the cognitive ameliorative effects of Esc in AD.

Oxidative stress, particularly that created by NOX induction, has been implicated to play a central role in the development of AD^58^. NOX activity is strongly correlated with cognitive performance such that increases in NOX enzyme activity decrease the cognitive function^59^. The heightened induction of NOX expression and activity in AD may be ascribed, in part, to the increased Aβ levels^60^. NOX has also been suggested to mediate cerebrovascular dysfunction induced by Aβ^61^. Furthermore, it has been reported that microglia mediate Aβ-induced neurotoxicity through induction of NOX^62^. In addition to playing a role in ROS generation, NOX has been proven to promote neuroinflammation and pro-inflammatory cytokine expression^63^ through activation of NF-κB^64^. Conspicuously, the present data revealed the ability of Esc to reduce NOX1 mRNA levels in D-gal/OVX-subjected rats, suggesting an antioxidant effect of Esc that may contribute partly to its neuroprotective effect in AD.

In the current investigation, treatment with Esc attenuated the D-gal/OVX-induced decreases in the gene expression of synaptophysin, a pre-synaptic protein; these results are in harmony with those of former studies^8,65^. Synaptic protein deficits occur very early in AD and are firmly linked to cognitive decline. These deficits are caused by Aβ aggregation and may eventually damage the neurons^66^. Furthermore, Esc treatment upregulated the hippocampal p-CREB protein expression and BDNF content in D-gal/OVX-subjected rats. CREB has been identified as a key element for learning and memory as well as for neuronal plasticity. Its phosphorylation is essential for transcriptional activation that leads to gene products such as BDNF^67^. BDNF, the most abundant neurotrophin in the hippocampus, also plays pivotal roles in synaptic plasticity and neuronal survival^68^. BDNF binds to its receptor, tyrosine receptor kinase B, to activate the PI3K/Akt and ERK1/2 pro-survival pathways^33,69^. Studies have reported altered levels of BDNF in the circulation of AD patients and low BDNF levels in the cerebrospinal fluid of healthy older subjects as predictors of future cognitive decline^68^. Further, improved learning and memory have been observed in genetically engineered mice overexpressing BDNF^70^. Thus, the enhanced p-CREB, BDNF, and synaptophysin levels along with the reduced NOX1 levels in this study might explain the salient neuroprotective effects of Esc. These effects were evidenced by the reduced number of degenerated pyramidal neurons in the hippocampal areas of Esc-treated rats.

Inflammation is a crucial event in the pathogenesis of AD. This process is driven by the activation of microglial and astrocytic cells, which produce a plethora of inflammatory mediators such as pro-inflammatory cytokines, chemokines, prostaglandins, ROS, and nitric oxide synthase. This activation may be due to a glial response to the ongoing deposition of Aβ^71^. Pro-inflammatory cytokines have detrimental effects on neurogenesis and cause further bystander damage to neurons^2^. Herein, the observed D-gal/OVX-induced elevation in hippocampal TNF-α was partially mitigated by Esc treatment, suggesting that Esc has anti-neuroinflammatory properties that could contribute to its neuroprotective role. In the current investigation, treatment with Esc also decreased hippocampal NF-κB p65 content and associated pathological events in D-gal/OVX-subjected animals. Activated NF-κB induces the transcription of NOX^72^, β-secretase^26^, and various genes involved in inflammation, including TNF-α^73^. Additionally, it reduces the capability of microglial cells to phagocytose Aβ42 peptide monomers, favouring their assembly into higher-order amyloid species^74^. Thus, the Esc-induced amelioration of the neuroinflammatory response may be attributable to an ability of Esc to inhibit Aβ, p-tau, NF-κB p65, NOX1, and GSK-3β.

## Conclusion

**Figure 7:**
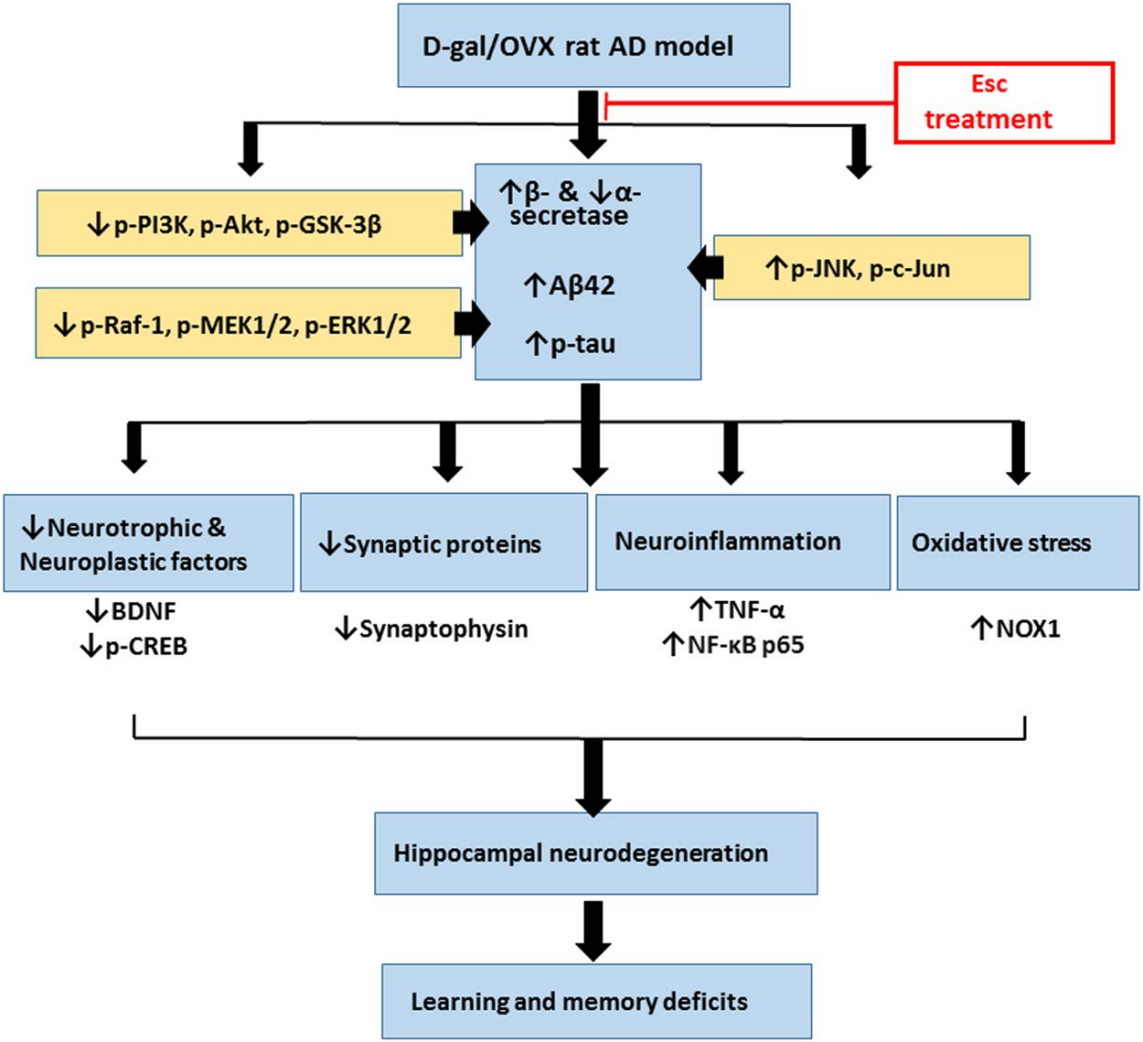
Diagram depicting the attenuation of the pathological aberrations induced in D-galactose/ovariectomized rats by escitalopram. *D-gal* D-galactose, *OVX* ovariectomy, *AD* Alzheimer’s disease, *Esc* escitalopram, *Aβ42* amyloid β 42, *p-tau* phosphorylated tau, *PI3K* phosphoinositide 3-kinase, *Akt* protein kinase B, *GSK-3β* glycogen synthase kinase-3β, *ERK* extracellular signal-regulated kinase, *JNK* c-Jun N-terminal kinase, *NOX* nicotinamide adenine dinucleotide phosphate oxidase, *BDNF* brain-derived neurotrophic factor, *p-CREB* phospho-cAMP response element binding protein, *TNF-α* tumor necrosis factor-α, *NF-κB p65* nuclear factor-kappa B p65.

The current study revealed that chronic Esc treatment ameliorated cognitive dysfunction and mitigated AD-like features in D-gal/OVX-subjected rats. Esc reduced Aβ and p-tau, hampered neuroinflammation and NOX-derived oxidative stress, and enhanced the levels of neurotrophic, neuroplastic, and synaptic proteins. These beneficial effects are proposed to be mediated through activation of PI3K/Akt/GSK-3β and Raf-1/MEK/ERK pathways and inhibition of JNK/c-Jun cascade (Fig. 7). Therefore, the present findings provide compelling evidence that Esc is a promising agent to halt AD progression. Further studies analysing transcriptomic and proteomic profiles in brain tissues are required in the future for providing more information regarding Esc-induced effects.

## Supplementary information

Supplementary file for Western blot images

## Data Availability

All data generated or analysed during this study are included in this article and its Supplementary Information Files.
