# Supplementary material for: Escitalopram Ameliorates Cognitive Impairment in D-Galactose-Injected Ovariectomized Rats: Modulation of JNK, GSK-3β, and ERK Signalling Pathways
Source: Sci Rep. 2019 Jul 11;9:10056. doi: 10.1038/s41598-019-46558-1 (PMC6624366; doi:10.1038/s41598-019-46558-1)

Escitalopram Ameliorates Cognitive Impairment in D-Galactose-Injected Ovariectomized Rats: Modulation of JNK, GSK-3β, and ERK Signalling Pathways

Weam W. Ibrahim [a,*], **Noha F. Abdelkader** [a], **Hesham M. Ismail** [b], **Mahmoud M. Khattab** [a]
[a] Department of Pharmacology & Toxicology, Faculty of Pharmacy, Cairo University, Cairo, Egypt.
[b] School of Pharmaceutical Sciences, University of Geneva, Geneva, Switzerland.

Corresponding author: Weam W. Ibrahim
E-mail: weam.wadie@pharma.cu.edu.eg

p-PI3K

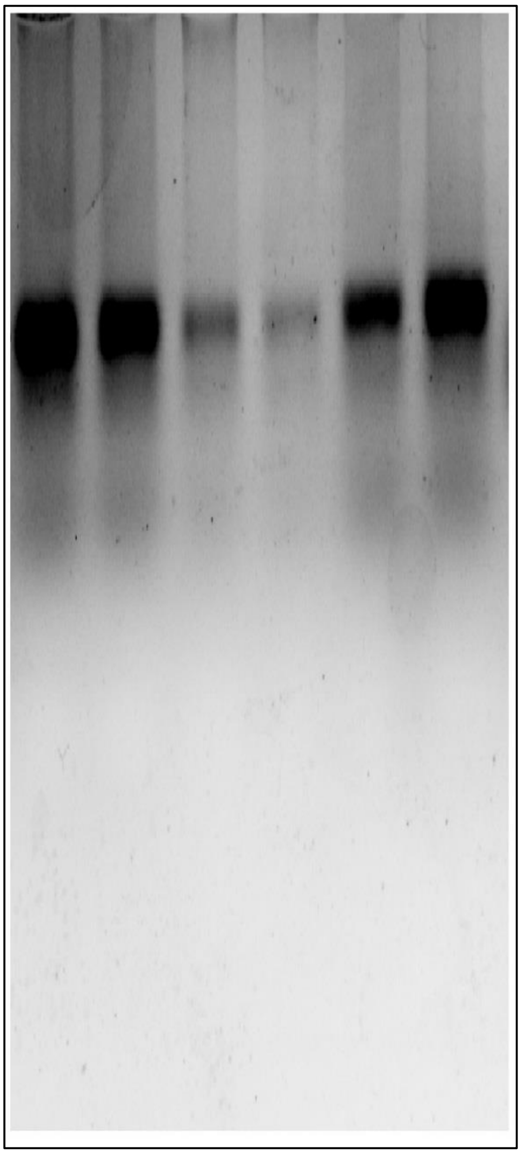

p-Akt

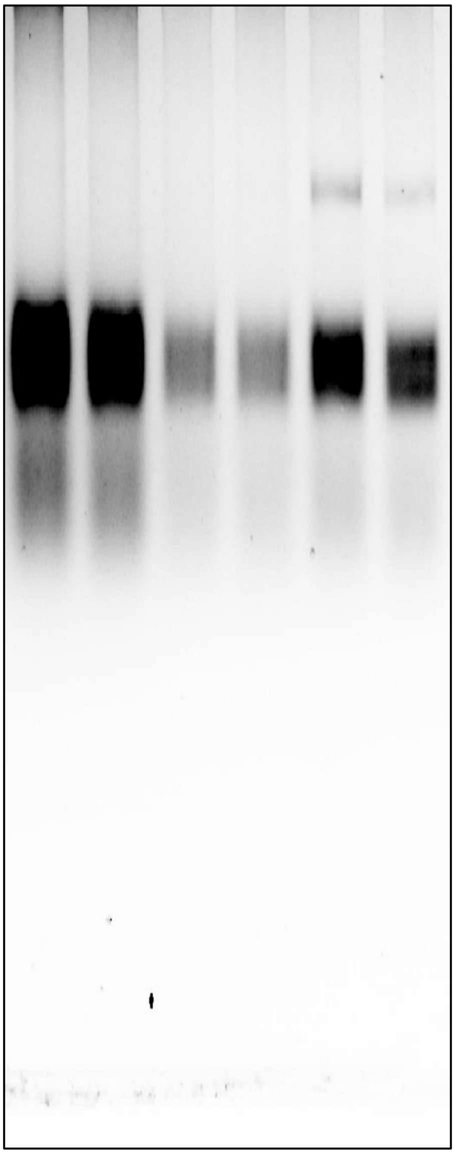

p-GSK-3β

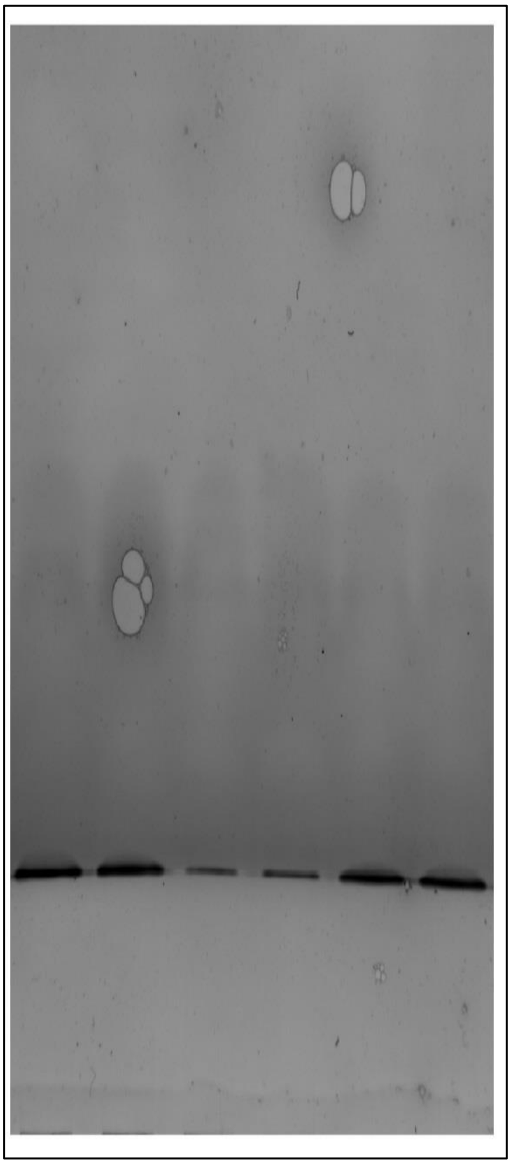

β-actin

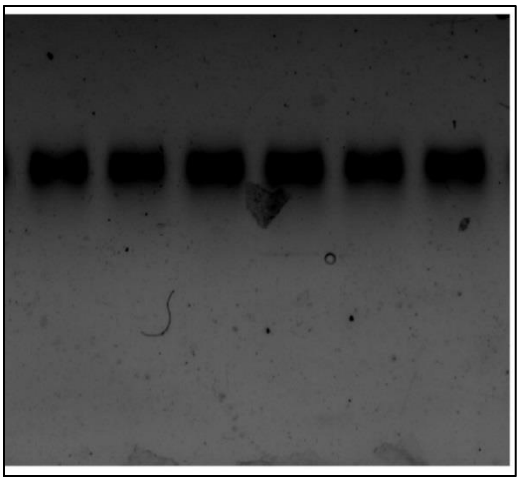

β-actin

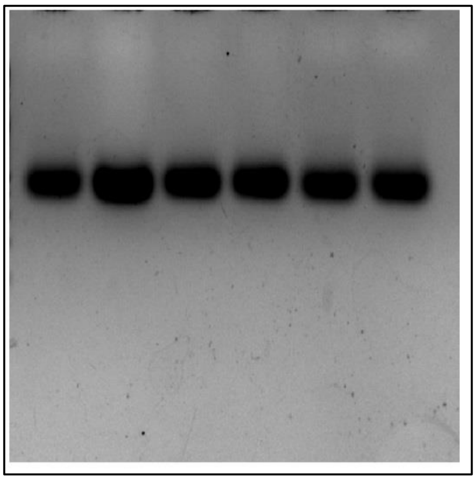

β-actin

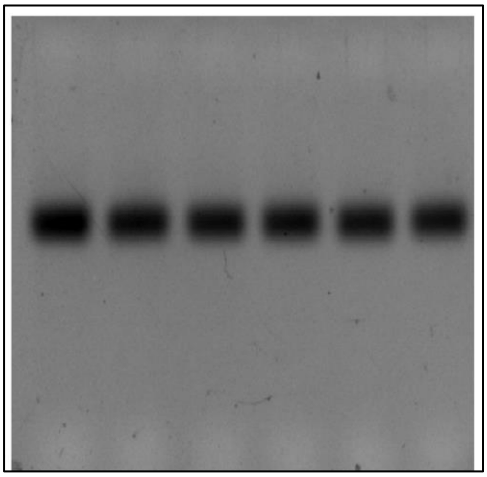

p-Raf-1

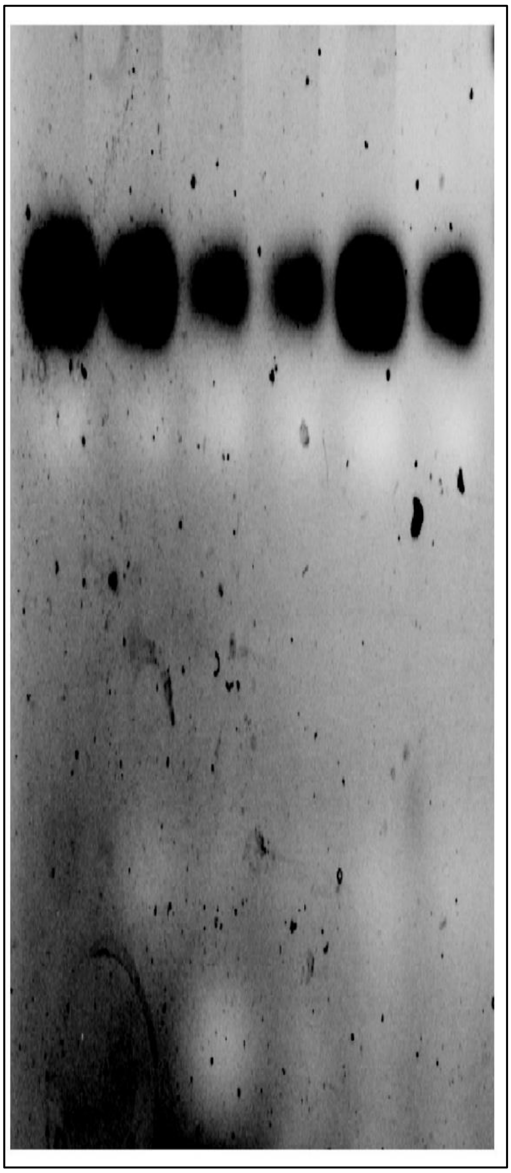

p-MEK1/2

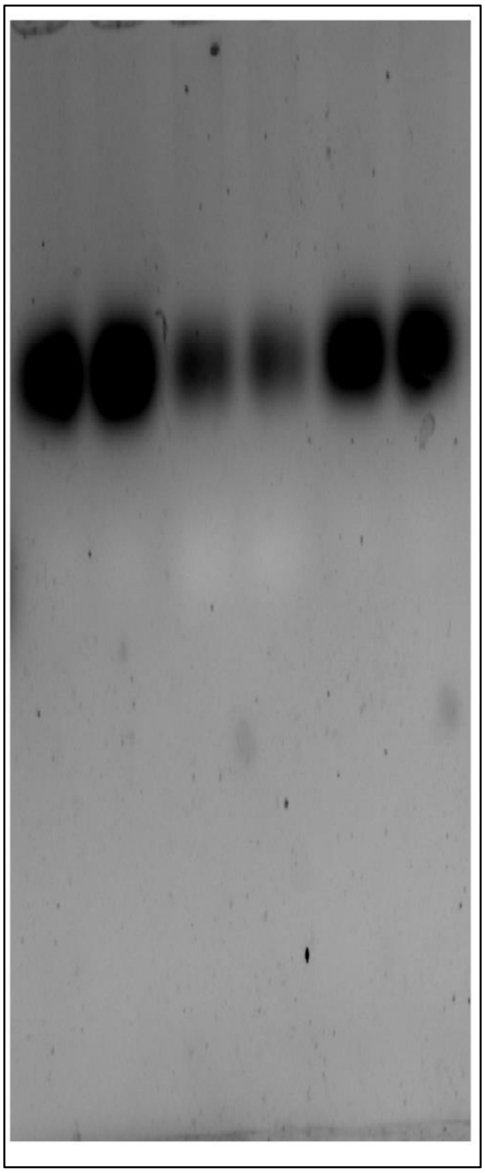

p-ERK1/2

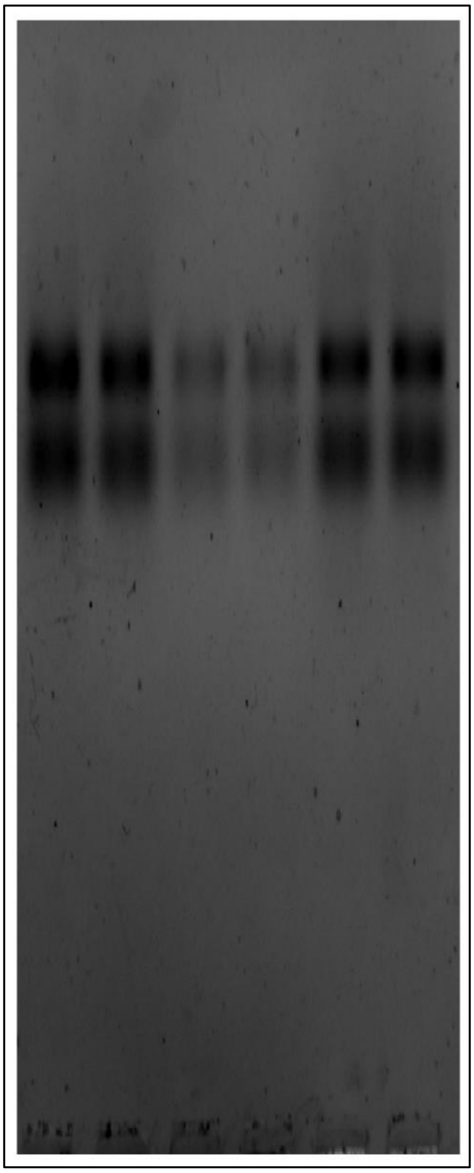

β-actin

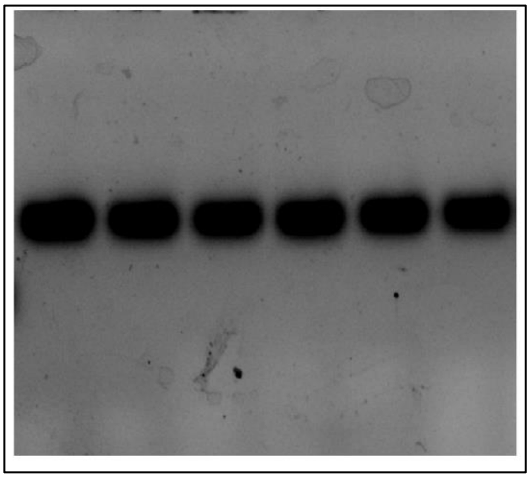

β-actin

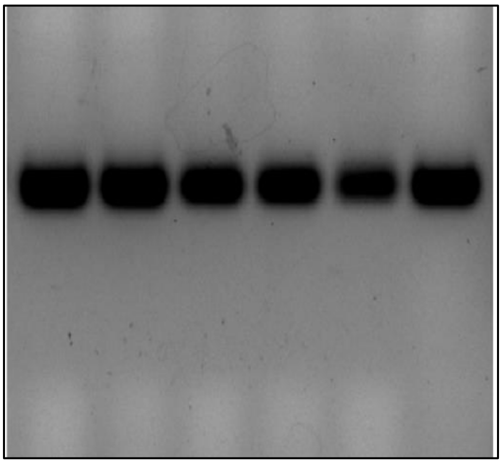

β-actin

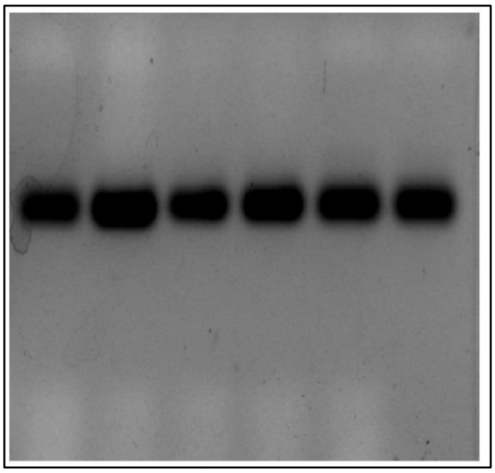

p-JNK

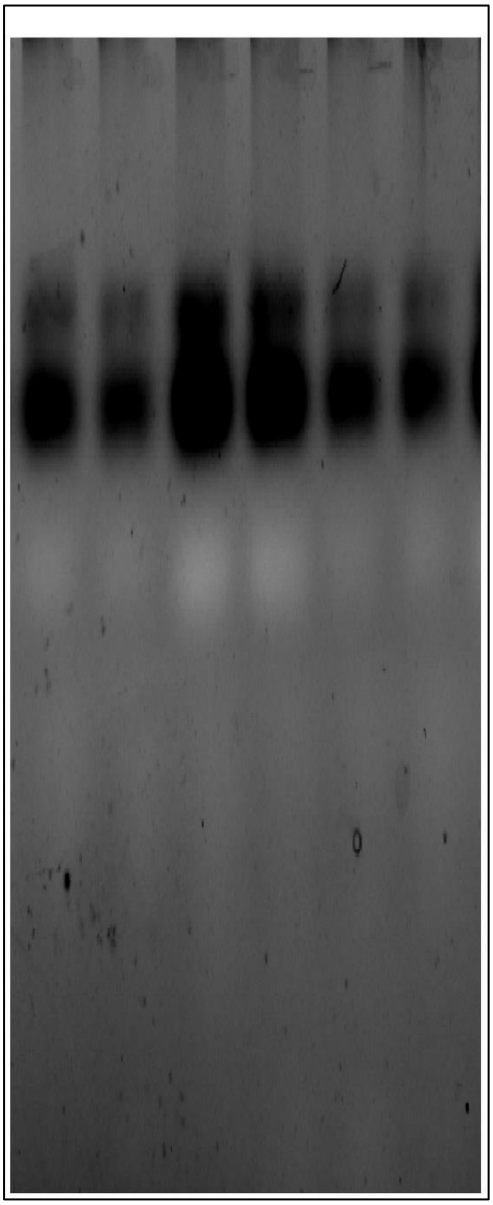

p-c-Jun

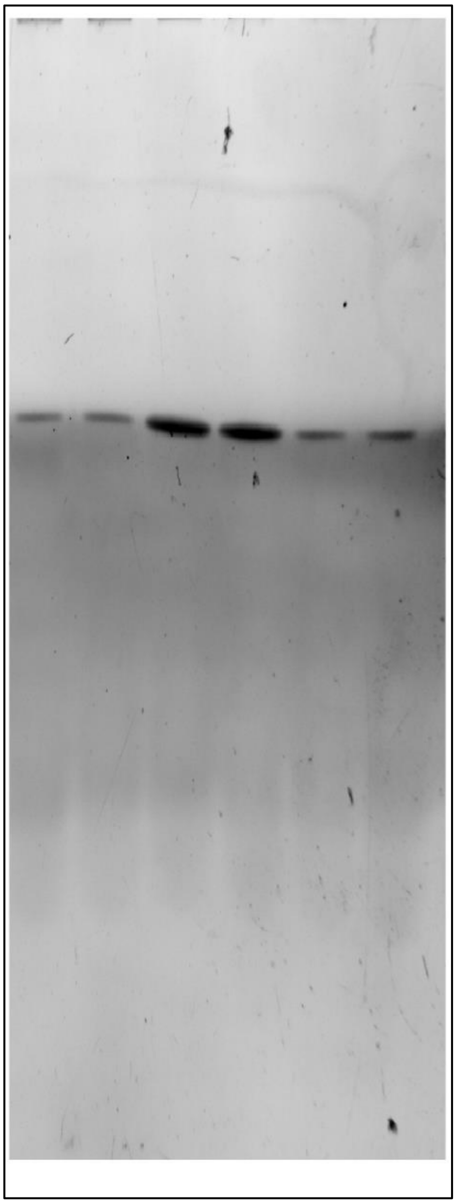

p-tau

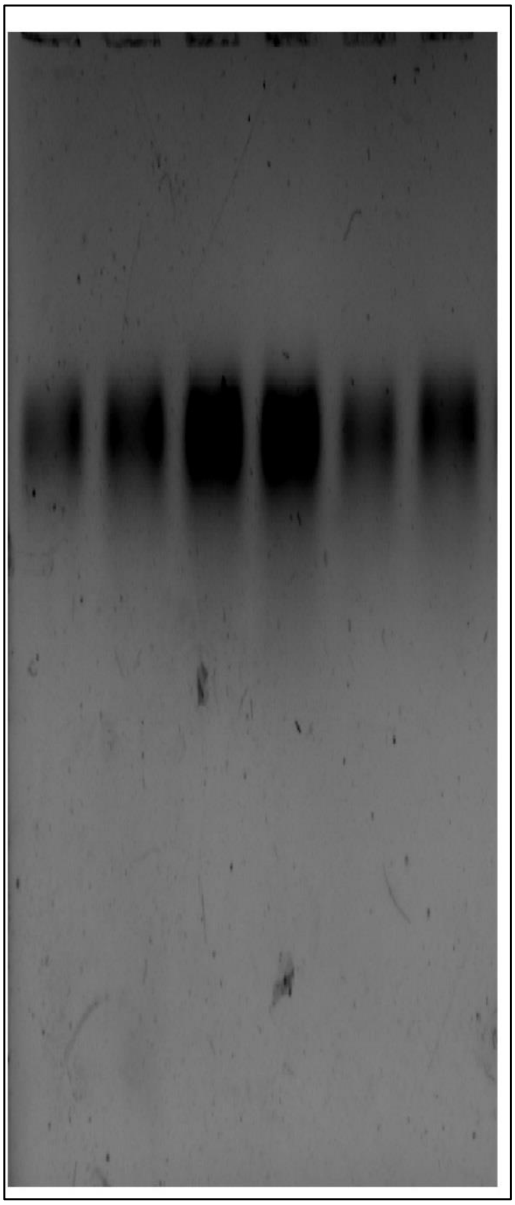

β-actin

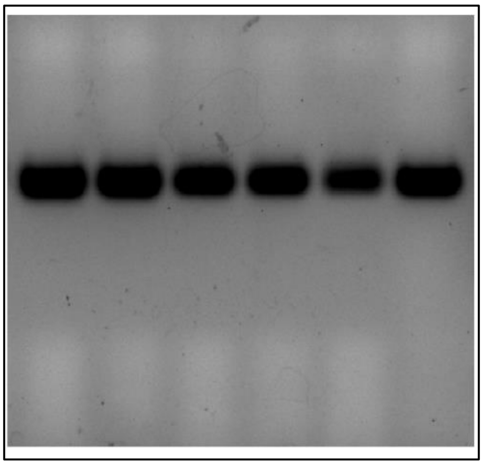

β-actin

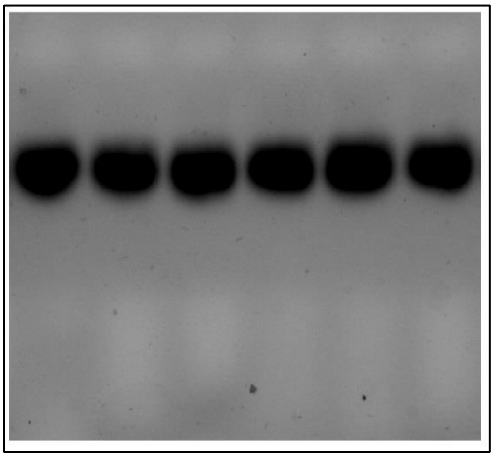

β-actin

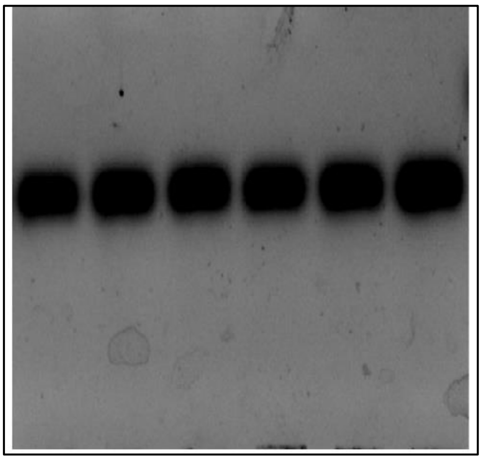

p-CREB

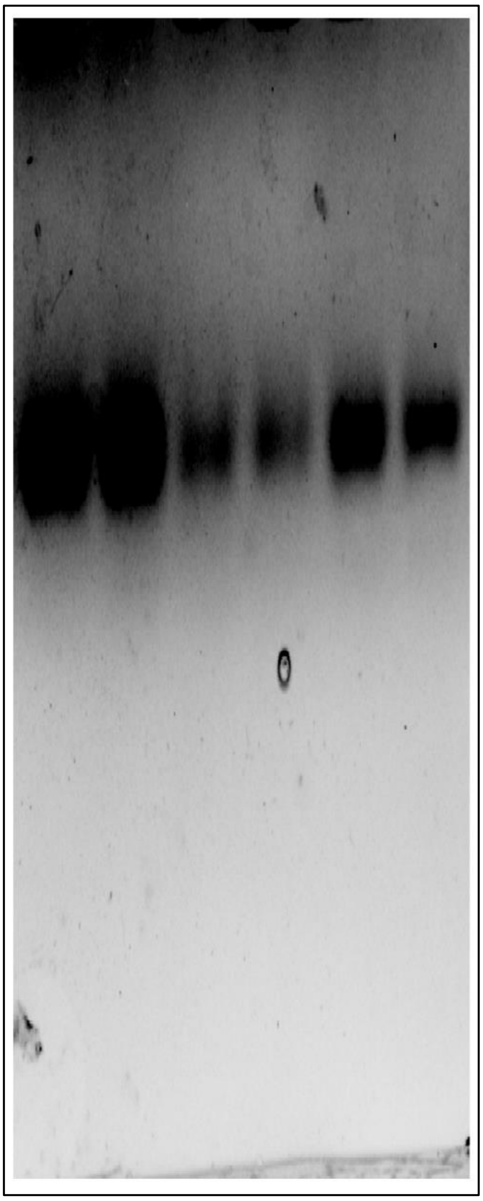

β-actin

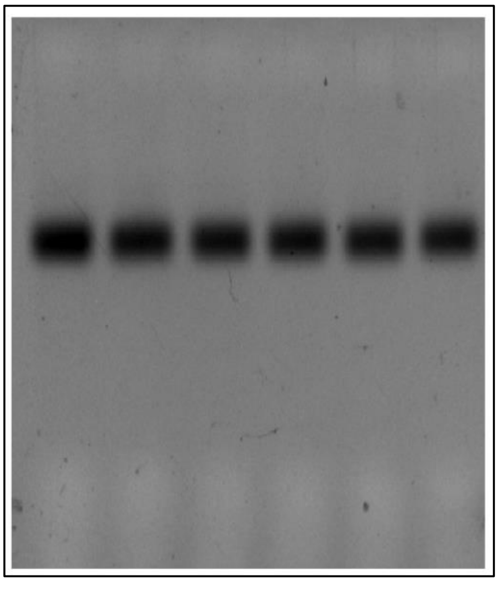

Supplement: Supplementary file 1 — Supplementary file for Western blot images [file 41598_2019_46558_MOESM1_ESM.pdf]
